# Acetylcholine and Acetylcholine Receptors: Textbook Knowledge and New Data

**DOI:** 10.3390/biom10060852

**Published:** 2020-06-03

**Authors:** Victor I. Tsetlin

**Affiliations:** Shemyakin-Ovchinnikov Institute of Bioorganic Chemistry, Russian Academy of Sciences, 117997 Moscow, Russia; victortsetlin3f@gmail.com

It was a pleasure to receive a proposal to organize and be a guest editor of a Special Issue of *Biomolecules.* This is the field in which I am working and personally know some of the leading scientists. My narrow field is the research on the peptide and protein neurotoxins from animal venoms and their application as sophisticated tools for analysis of nicotinic acetylcholine receptors (nAChRs). Recently, together with my colleagues from several European laboratories, I was a guest editor for the Special Issue of *Frontiers in Pharmacology,* devoted to diverse neurotoxins acting on various ligand-gated or voltage-gated ion channels. In this Special Issue, it was decided to focus mostly on the nAChRs. The word “acetylcholine” in the title of the issue is due to the interest in the mechanism of action of this naturally-occurring agonist and also in other endogenous or synthetic compounds regulating nAChR functions and having the potential to lead to novel drugs. Since there are examples where a compound acts on different targets, like galanthamine which inhibits acetylcholinestrase, but is a positive allosteric modulator of the α7 nAChR, or some non-conventional snake venom neurotoxins interacting with both nAChRs and with muscarinic receptors, we decided to cover, although with less detail, muscarinic acetylcholine receptors.

The words in the title “textbook knowledge and new data” perfectly agree with the content of papers published in this Special Issue. The paper by Prof. J.-P. Changeux [1] is not a modern review, which normally would be confined to recent data, but is, in essence, a chapter in a textbook telling the long history of nAChR, the first neurotransmitter receptor discovered, with details of its isolation, structural characterization, cloning, and elucidation of its mechanisms of action. In the presented multitude of approaches applied in nAChR research, is the description of the crucial role played by snake venom protein neurotoxins in the first isolation and characterization of nAChR; at present, together with the α-conotoxin neuropeptides, they are still playing a role as sophisticated tools in distinguishing various muscle and neuronal nAChRs subtypes. Involvement of the muscle-type nAChRs in diseases became clear with the disease myasthenia gravis, while at present the neuronal nAChRs are considered as drug targets against nicotine dependence and neurodegenerative diseases.

This paper is especially interesting, because the nAChR history is described by Prof. Changeux, who has over 50 years in this field and has made many discoveries. In particular, one of his activities is described in the sub-chapter “The first visualization of the receptor protein structure by electron microscopy”. After this N. Unwin, one of the fathers of cryo-electron microscopy, had to spend about 15 years to determine the structure of the *Torpedo marmorata* nAChR with the resolution of about 4A [2]. Since Prof. Changeux’s paper stressed the immense role played by the *Torpedo marmorata* nAChR and α-bungarotoxin in the whole research on the nAChRs, it is appropriate to mention a paper which has just appeared and has presented a high-resolution (2.7 A) cryo-electron microscopy structure of the complex between α-bungarotoxin and *Torpedo californica* nAChR [3]. It is also appropriate to mention that Prof. Changeux, in his detailed review, reflected the role of his school. However, from the very beginning, nAChR research has attracted many of the best scientists from all over the world. At the initial stage of structural characterization of the *Torpedo* nAChR and in elucidating the topography of the binding site and of the channel moiety, a very important role was played by Prof. Arthur Karlin (Cornell University, USA) who is a co-author of the above-mentioned paper on the α-bungarotoxin-nAChR complex [3].

Structural analysis results are even more impressive for the muscarinic acetylcholine receptors where 3D structures were determined for all M1–M5 subtypes, and in this issue we have a review by Jakubik and El-Fakahany [4] devoted to one particular problem, namely the allosteric modulation of muscarinic receptors. Interestingly, allosteric modulation is also an important topic for nAChRs and it is appropriate to remind readers that the concept of allosterism for enzymes was introduced first by Monod, Wyman, and Changeux [5] and later was applied most successfully to receptors in the case of nAChRs. The review on muscarinic receptors provided detailed information on the chemical structure of allosteric modulators, on the location of their binding sites, and on their disposition relative to the binding centers for the orthosteric ligands. In combination with the “bitopic” ligands, these compounds are considered as possible routes to drugs against neurological, psychiatric, and neurodegenerative diseases.

All other papers in this issue present experimental results concerning the activity of distinct nAChR subtypes, in order to gain more information on their functioning and to find the ways to new drugs. Much attention is attracted by the α7 nAChRs due to their functional role both in the brain and in the immune system. Working with them is not easy because of problems with their expression and fast desensitization. Deshpande et al. [6] discuss why knock-outs of such chaperons as Ric3 and NACHO differently affect the expression of the α7 nAChRs.

Concerning the role of distinct nAChR subtypes as targets for various drugs, it is appropriate to mention the paper by Qui et al. [7] where it was shown that knock-out of the α10 nAChR subunit in the model of experimental autoimmune encephalomyelitis (EAE) does not grossly change the situation, thus providing further evidence of the disease-exacerbating roles for nAChR containing α9 subunits in EAE inflammatory and autoimmune responses.

In two papers the effects of unusual ligands were tested on different nAChR subtypes. Akimov et al. [8] found that cholines acylated with unsaturated fatty acids (arachidonoyl choline and others), a recently-discovered family of endogenous lipids, behaved as inhibitors of the muscle-type and α7 neuronal nAChR. Lykhmus et al. [9] checked what could be the effects of N-stearoylethanolamine (NSE), a lipid possessing anti-inflammatory, cannabimimetic, and membrane-stabilizing activity, on brain mitochondria sustainability in mice where the α7 nAChRs were knocked-out. This compound stimulated the rise of interleukin-6 in the blood and improved episodic memory of α7-/- mice. NSE improved the brain mitochondria sustainability to apoptogenic stimuli and up-regulated α4β2 nAChRs in the brain. It was concluded that the level of alternative nAChR subtypes in the brain is critically important for memory and mitochondria sustainability in the absence of α7 nAChRs.

The role of α7 nAChRs in the cholinergic anti-inflammatory pathway is well known and was earlier shown to affect the expression of several cytokines. Siniavin et al. [10] analyzed it in more detail using α7 nAChR selective agonist PNU 282,987. They confirmed the effects on the expression of such cytokines as IL-6 and IL-10, but for the first time demonstrated the changes in the expression of such membrane molecules as HLA-DR, CD54, CD11b, and CD14 which opens new ways to prevent immunosuppression.
